# The Neuroprotective Role of Indole-3-Propionic Acid in Migraine Pathophysiology

**DOI:** 10.3390/medicina60091417

**Published:** 2024-08-30

**Authors:** Dilek Agircan, Seyhan Taskin, Murat Cekic, Hakim Celik

**Affiliations:** 1 Department of Neurology, Harran Faculty of Medicine, Harran University, Sanliurfa 63290, Turkey; cekicdr@gmail.com; 2 Department of Physiology, Harran Faculty of Medicine, Harran University, Sanliurfa 63290, Turkey; seyhan_taskin@yahoo.com (S.T.); hakimcell@gmail.com (H.C.)

**Keywords:** migraine, indole-3-propionic acid, nitrosative stress, pain severity, disability

## Abstract

*Background and Objectives*: Migraine is a leading cause of disability worldwide, with complex pathophysiological mechanisms involving oxidative and nitrosative stress. Recent research suggests that Indole-3-Propionic Acid (IPA) may have a neuroprotective role in reducing nitrosative stress. This study aims to elucidate the roles of IPA and nitrosative stress biomarkers in migraine patients, focusing on their potential as therapeutic targets. *Materials and Methods*: This cross-sectional, case-control study included 57 migraine patients and 30 healthy controls. Patients were categorized into episodic migraine (EM) and chronic migraine (CM) groups. Socio-demographic and clinical characteristics were documented through structured interviews. Validated scales such as the Visual Analog Score (VAS), Headache Impact Test 6 (HIT-6), Migraine Disability Assessment Test (MIDAS), Migraine 24 h Quality of Life Scale (24 h QoL), Mini-Mental State Examination (MMSE), and Migraine Attacks–Subjective Cognitive Impairments Scale (Mig-SCog) were administered. Venous blood samples were collected, and serum levels of IPA, Nitric Oxide (NO), Nitric Oxide Synthase (NOS), and Peroxynitrite (ONOO^−^) were measured using ELISA and spectrophotometric methods. *Results*: Significant differences in serum IPA and NO levels were observed between migraine patients and controls. Specifically, higher serum IPA levels were found in the EM group, while higher serum NO levels were observed in the CM group. Elevated NO levels correlated with increased migraine attack frequency. Conversely, serum IPA levels showed a negative correlation with attack frequency, suggesting a protective role. Specifically, NO levels were positively correlated with the number of painful days, NSAID usage, VAS scores, HIT-6 scores, and MIDAS scores, while negatively correlated with 24 h QoL scores. *Conclusions*: The study highlights the significant involvement of IPA and nitrosative stress in migraine pathophysiology. Elevated IPA levels, particularly in EM patients, suggest its potential neuroprotective role. These findings underscore the importance of targeting oxidative and nitrosative stress pathways in developing effective migraine therapies.

## 1. Introduction

According to the Global Burden of Disease Study 2016, migraine ranks as the sixth leading cause of disability worldwide [1]. Migraines are classified into CM and EM. EM is defined as experiencing up to 14 headache days per month, whereas CM is defined as having 15 or more headache days per month [2,3]. Without appropriate treatment, EM can progress to CM over time [4].

Despite extensive research, the pathophysiological processes underlying migraine remain incompletely understood, though it is known to result from the interplay of genetic, environmental, and neurological factors. Studies have elucidated that migraine is a neurovascular disorder associated with mechanisms such as the hyperactivation of pain pathways in the brain, activation of the trigeminal nerve system, neurogenic inflammation, and vasodilation [5]. During inflammation, activated phagocytic cells such as neutrophils and macrophages generate significant amounts of reactive oxygen, nitrogen, and chlorine species to eliminate pathogens, but excessive production of these reactive species can escape the phagocytic cells and cause localized oxidative stress and tissue damage [6]. Various studies have indicated that oxidative and nitrosative stress could be involved in the biological processes underlying migraine [7,8]. Oxidative stress occurs when there is an imbalance between the production and degradation of reactive oxygen species (ROS) or reactive nitrogen species (RNS). ROS are highly reactive molecules that originate from the metabolism of oxygen or nitrogen. Both ROS and RNS can exist as free radicals, including superoxide (O^2−^), hydroxyl (OH), or nitric oxide (NO^•^). Additionally, non-free radical forms such as hydrogen peroxide (H_2_O_2_) and peroxynitrite (ONOO^−^) can also be present [9]. This complex interaction underscores the need for further research to elucidate the pathogenesis of migraine and develop effective therapeutic strategies.

The relationship between tryptophan (TRP) metabolism and migraine has significantly contributed to a better understanding of the complex pathophysiology of migraine in recent years [10]. TRP is metabolized primarily through three pathways: approximately 95% via the kynurenine pathway, around 1–2% for serotonin synthesis, and the remainder is metabolized by the gut microbiota to produce indoles and related derivatives [11,12,13]. The presence of serotonin receptors in the trigeminal system and cranial blood vessels has been documented, and agonists for these receptors, including triptans, are effective in alleviating migraines. Thus, serotonin-mediated neurotransmission is implicated in migraine mechanisms. The kynurenine pathway generates neuroactive metabolites that influence trigemino-vascular activation processes and interact with glutamate receptors in the central nervous system (CNS), potentially playing a role in the pathophysiology of migraine [10]. However, research on the indole pathway, the other metabolic route of tryptophan, remains limited.

In the indole pathway, TRP is initially converted to indole-3-pyruvic acid, which subsequently transforms into indole-3-lactic acid and then through anthranilic acid to produce indole-3-propionic acid (IPA) [14,15]. IPA has been found to exhibit various pharmacological activities, including anti-inflammatory, antioxidant, and neuroprotective effects [16,17,18]. Treatment with IPA significantly inhibited the increase in inducible nitric oxide synthase (NOS) in the hypoxic-ischemic brain injury model [19]. It has been speculated in the literature that IPA negatively affects the physiological control of vascular tone by impairing the endothelial NO^•^ release induced by purinergic stimulation [20]. We hypothesized that IPA, known for its anti-inflammatory effects, can reduce nitrosative stress in migraine. Our primary objective is to determine the levels of IPA, NO^•^, NOS, and ONOO^−^ in migraine patients to elucidate their role in migraine pathophysiology and assess their therapeutic potential.

## 2. Material and Methods

### 2.1. Study Design

Compared to a control group, this cross-sectional, case-control study is designed to investigate the levels of IPA, NO^•^, NOS, and ONOO^−^ in patients with migraine. All patients included in this research were treated as outpatients at the Department of Neurology, Harran University, Faculty of Medicine, and provided written informed consent before their inclusion in the study. The study received approval from the local Ethics Committee of Harran University (HRU/24.07.34). The study followed the principles outlined in the Declaration of Helsinki.

### 2.2. Participants

The study included 18–65-year-old patients, comprising 57 patients with migraine who met the criteria from the third edition of The International Classification of Headache Disorders [21] and 30 healthy control participants. Based on these criteria, the patient group was subsequently categorized into those with episodic migraine (EM) and those with chronic migraine (CM). The control group was selected from volunteers with comparable demographic characteristics and no history of any illnesses. Participants were excluded if they had experienced any other type of headache, had a history of autoimmune or inflammatory diseases, had undergone any major surgical procedures within the last six months, had a history of malignancy or cancer treatment in the past five years, or were using any chronic medication. The patients were not placed on exogenous IPA or any related supplements.

### 2.3. Data Collection

After meeting the inclusion and exclusion criteria, the same neurologist conducted a structured interview. During the initial visit, socio-demographic and clinical characteristics were documented, such as age, gender, body mass index (BMI), migraine characteristics (frequency and type), and medication overuse. Several validated scales including the Visual Analog Score (VAS) [22] to quantify pain severity, Headache Impact Test 6 (HIT-6) [23] to measure the impact of headaches on daily life, the Migraine Disability Assessment Test (MIDAS) [24] to evaluate the disability caused by migraine, the 24 h Quality of Life (24 h QoL) Scale in Migraine [25] to assess the impact of migraine on quality of life over 24 h, the Mini-Mental State Examination (MMSE) [26] to assess cognitive function, and Migraine Attacks–Subjective Cognitive Impairments Scale (Mig-SCog) [27] to assess cognitive impairments related to migraine attacks were recorded.

### 2.4. Blood Sample Collection

The study was conducted over a one-month period, with venous blood samples collected once during the patient’s initial visit. A venous blood sample (approximately 10 mL) was collected from each participant using standard venipuncture techniques after an overnight fast. The blood samples were centrifuged at 3000 rpm for 10 min to separate the serum. The serum was aliquoted into separate tubes and stored at −80 °C until analysis.

### 2.5. Biochemical Analysis

#### 2.5.1. Measurement of Indole-3-Propionic Acid (IPA)

Serum IPA (Cat.No: MBS3804616) levels were determined using a commercial ELISA kit following the manufacturer’s instructions (MyBiosource, Inc., San Diego, CA, USA). The kit works on the competitive ELISA principle. The micro-ELISA plate included in the kit is pre-coated with an antibody specific to human IPA. Serum samples and IPA Standards are added to the micro-ELISA plate wells and combined with the specific antibody. Then, HRP-labeled human IPA antigen is added. Unreacted components are removed by washing. After washing, only the antibody enzyme-labeled antigen immune complex will remain on the solid microplate surface. After adding Chromogen Solution A and Chromogen Solution B, the immune complex will change to blue by HRP catalyzing. The reaction is terminated by adding a stop solution and the color turns yellow. The absorbance was measured with a microplate reader (Varioskan LUX; Thermo Fisher Scientific, Waltham, MA, USA) at a wavelength of 450 nm. The concentration of IPA in the serum is then determined by comparing the O.D. of the samples to the standard curve. Serum IPA level was expressed as ng/mL.

#### 2.5.2. Determination of Nitric Oxide (NO^•^) Level

The nitric oxide levels were analyzed by measuring total nitrite/nitrate concentrations (stable end-products of NO^•^) in the serum using diazotization or the Griess assay method [28,29]. The method’s basic principle is based on the formation of azo compounds by the reaction of nitrite, an indirect indicator of NO^•^, with α-naphthylamine (Sigma, St. Louis, MO, USA) and sulfanilic acid (Sigma, St. Louis, MO, USA) at an acidic pH. Unmeasured nitrate in the reaction medium is reduced to nitrite with cadmium (Merck, Darmstadt, Germany) for a complete analysis. The NO^•^ levels are calculated by comparing the absorbance of the azo chromogen solution to a calibration curve prepared with known sodium nitrite concentrations (1–100 µmol/L). NO^•^ level was expressed as umol/L.

#### 2.5.3. Determination of Peroxynitrite (ONOO^−^) Level

The peroxynitrite assay modified by Al-Nimer et el. was determined as previously described [30,31,32]. In the first step of the two-stage experiment, to obtain a final volume of 2 mL, 10 µL of serum was mixed with 5 mM phenol (Sigma, St. Louis, MO, USA) and 600 mM Angeli’s Salt (Merck, Darmstadt, Germany) in a 50 mM sodium phosphate buffer. The mixture was incubated for 2 h at 37 degrees in the dark, and then 15 µL of 0.1 M NaOH (Merck, Darmstadt, Germany) was added. After incubation, absorbances were measured with a microplate reader (Varioskan LUX; Thermo Fisher Scientific, Waltham, USA) at a wavelength of 412 nm. The yield of nitrophenol was calculated from ε = 4400 M^−1^·cm^−1^. ONOO^−^ level was expressed as umol/L.

#### 2.5.4. Nitric Oxide Synthase (NOS) Activity Assay

NOS activity was determined as previously described [33,34]. First, NOS substrate L-arginine was added to the serum sample and incubated at 25 °C for 1 h. Following incubation, for the diazotization reaction, the combination of reactives was added. After the second incubation of 10 min, the absorbance of the sample tube was measured spectrophotometrically at 540 nm against a blank tube to which no arginine (Sigma, St. Louis, MO, USA) was added. The sodium nitroprusside (Sigma, St. Louis, MO, USA) was used as the chemical standard. The results are expressed as U/mL.

### 2.6. Statistical Analysis

SPSS statistical software version 24 (IBM Inc., Triangle Park, NC, USA) was used to perform all statistical analyses. The standard data distribution was tested with the Shapiro–Wilk test. Numerical variables that followed a normal distribution were presented as means ± standard deviations, whereas those not conforming to normal distribution were described using median and interquartile range values. Categorical variables were expressed as numbers and percentages while compared with the Pearson Chi-square and Fisher exact tests. Differences between the two groups were tested using either the Mann–Whitney U test or the independent samples *t*-test, depending on the parametric or non-parametric nature of the data. For comparisons involving three or more groups, one-way Analysis of Variance (ANOVA) or Kruskal–Wallis tests were employed, based on whether the data were parametric or non-parametric. The Spearman or Pearson rank correlation was utilized for the correlation analysis, depending on the parametric or non-parametric nature of the data. A *p*-value < 0.05 was assumed to be statistically significant, and all *p*-values were based on two-sided testing.

## 3. Results

The study included 87 participants, categorized into two groups: 30 controls and 57 patients. There were no significant differences in age, gender distribution, or body mass index (BMI) among the groups, ensuring a homogenous sample for comparison. The demographic and biochemical results between the patients and the control group are presented in Table 1.

The subgroup analysis compared the demographic and biochemical characteristics of the control, episodic, and chronic groups. The patient group was subdivided into 28 patients with EM and 29 with CM. Key variables analyzed included age, gender, BMI, serum IPA, NOS, NO^•^, and ONOO^−^ levels reflecting the body’s intrinsic production without any influence from exogenous supplementation. Statistically significant differences were observed in IPA and NO^•^ levels across the groups, with *p*-values of 0.001 for both variables (Table 2).

Notable findings included higher attack frequency, number of painful days, and NSAID usage in the CM group compared to the EM group. Additionally, significant differences were observed in VAS, HIT-6, MIDAS, 24 h QoL, Mig-SCog, IPA, and NO^•^ levels, with *p*-values indicating strong statistical significance. Detailed comparative results are provided in Table 3.

**Figure 1:**
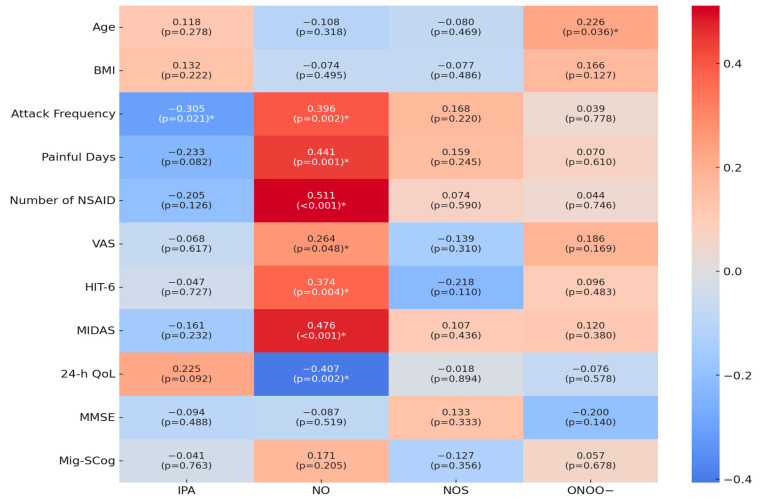
Correlation Analysis of IPA and Nitrosative Stress Parameters with Clinical Variables and Measurement Scales. Abbreviations: BMI = Body Mass Index, NSAID = Non-Steroid Anti-Inflammatory Drug, VAS = Visual Analog Score, HIT-6 = Headache Impact Test 6, MIDAS = Migraine Disability Assessment Test, 24 h QoL = 24 h Quality of Life, MMSE = Mini-Mental State Examination, Mig-SCog = Migraine Attacks–Subjective Cognitive Impairments Scale, IPA = Indole-3-propionic acid, NO^•^ = Nitric Oxide, NOS = Nitric Oxide Synthase, ONOO^−^ = Peroxynitrite. * Statistically significant *p*-values.

The correlations between various parameters, including serum IPA, NO^•^, NOS, and ONOO^−^ levels, and several other clinical and demographic variables are shown in Figure 1. A significant negative correlation was found between serum IPA levels and attack frequency (r = −0.305, *p* = 0.021). NO^•^ exhibited multiple significant correlations: it was positively correlated with the attack frequency (r = 0.396, *p* = 0.002), painful days (r = 0.441, *p* = 0.001), number of NSAIDs used (r = 0.511, *p* < 0.001), VAS scores (r = 0.264, *p* = 0.048), HIT-6 scores (r = 0.374, *p* = 0.004), and MIDAS scores (r = 0.476, *p* < 0.001). Furthermore, NO^•^ was negatively correlated with 24 h QoL scores (r = −0.407, *p* = 0.002). ONOO^−^ was positively correlated with age (r = 0.226, *p* = 0.036).

## 4. Discussion

The present study highlights the significant involvement of IPA and nitrosative stress in migraine pathophysiology. Our results indicate that the intrinsic production of both nitrosative stress and IPA levels was markedly elevated in the migraine groups compared to healthy controls, with particularly higher IPA levels in patients with EM. This observation supports that IPA may play a neuroprotective or compensatory role in migraine mechanisms.

Recent studies have indicated that patients suffering from CM and EM exhibit elevated levels of pro-inflammatory cytokines, such as C-reactive protein (CRP) and tumor necrosis factor alpha (TNF-α), compared to healthy individuals. Moreover, the literature underscores that higher concentrations of these inflammatory markers are associated with an increased likelihood of headaches transitioning to a chronic state [35]. The progression of many inflammatory diseases is driven by the central role of ROS production. RNS induces nitrosative stress, further contributing to the pro-inflammatory burden caused by ROS [36].

Oxidative stress results in an imbalance between the generation and elimination of ROS, which underlies the pathogenesis of numerous diseases [37]. At the onset of a migraine, markers indicating oxidative stress levels, such as malondialdehyde and total oxidant status, are elevated, while markers reflecting antioxidant capacity, including total thiol and glutathione, are diminished [38]. Reactive oxygen and nitrogen species generated during the oxidative stress associated with migraines can inflict damage on cellular macromolecules such as Deoxyribonucleic Acid (DNA), Ribonucleic Acid (RNA), proteins, and lipids [39]. Therefore, using antioxidants may help treat or alleviate migraine-like symptoms [40]. Oxidative stress was indicated by a rise in ROS levels during the onset in experimental animals in an animal model of migraine. After the administration of migraine-specific therapeutic drugs, these levels gradually decreased [41].

One of the pathophysiological mechanisms of migraine is known to be the excessive production of NO^•^ [42]. Gruber et al. suggest that individuals with migraines experience continuous nitrosative stress even during headache-free periods, speculating a non-linear relationship between NO^•^ levels and migraine occurrence. Elevated nitrosative stress during headache-free intervals is a significant risk factor for migraines [43]. In the literature, increased headache frequency is associated with elevated NO^•^ levels, alongside reduced antioxidant defenses, evidenced by lower antioxidant capacity values and decreased serum levels of superoxide dismutase and catalase. CM patients exhibited lower levels of total non-enzymatic antioxidant capacity and higher oxidative stress compared to EM patients and healthy controls [44].

IPA is known for its anti-inflammatory, antioxidant, and neuroprotective effects, which may mitigate the neuroinflammation and oxidative stress linked to migraines [16,17,18]. Due to its physiological presence in normal human plasma and cerebrospinal fluid, along with its strong antioxidant properties that lack pro-oxidant effects, its ability to inhibit amyloid-beta (Aβ) fibril formation, and its neuroprotective effects, IPA is considered a promising candidate for the development of Alzheimer’s disease therapy [45,46,47]. In Parkinson’s patients, plasma levels of serum IPA were significantly higher compared to controls [48]. In patients with relapsing–remitting multiple sclerosis who were within 30 days of a clinical relapse, urinary concentrations of IPA were significantly higher compared to those in clinically stable relapsing–remitting multiple sclerosis patients [49]. The independent predictive ability of serum IPA levels for acute cerebral infarction suggests its clinical utility in evaluating the progression and prognosis of stroke in acute cerebral infarction patients [50]. Studies have indicated that IPA promotes the regeneration and functional restoration of sensory axons through mechanisms involving the immune system [51]. In a mouse model of experimental autoimmune encephalomyelitis, IPA inhibited CNS inflammation in astrocytes [52]. Additionally, IPA treatment significantly inhibited the increase in inducible NOS expression in hypoxic-ischemic injury [53]. In the brain, IPA has been shown to mitigate neuronal damage and oxidative stress and influence inflammation and immunity [54]. The hormesis theory suggests that low doses of oxidative stress can induce adaptive and protective responses, while high doses predominantly cause harmful effects. At low doses, oxidative stress activates cellular antioxidant defense systems, making cells more resilient to future oxidative challenges. However, at high doses, these defense mechanisms may become overwhelmed, leading to cellular damage, impaired functions, and the development of various diseases [55]. We speculate that the higher IPA levels in the EM group compared to the CM group may result from the differential severity of nitrosative stress and inflammation. In EM, lower nitrosative stress and inflammation allow the endogenous antioxidant system to upregulate IPA, effectively managing oxidative stress. In contrast, severe nitrosative stress and inflammation in CM overwhelm the antioxidant system, preventing adequate IPA upregulation and contributing to persistent oxidative and nitrosative imbalance.

### Limitations

This study has several limitations that should be acknowledged. First, the cross-sectional design limits the ability to infer causality between elevated biochemical markers and migraine pathophysiology. Second, the sample size, while adequate for initial findings, may not be large enough to generalize the results to the broader population of migraine sufferers. Lastly, biochemical analyses were performed on serum samples, which may not fully capture the dynamic changes occurring within the central nervous system. Future studies with larger, more diverse populations and longitudinal designs are needed to confirm these findings and explore the causal relationships between nitrosative stress, IPA levels, and migraine pathophysiology.

## 5. Conclusions

In conclusion, this study underscores the significant role of IPA and nitrosative stress in the pathophysiology of migraines. Elevated levels of IPA were observed in migraine patients, particularly those with EM, suggesting a potential neuroprotective or compensatory function of IPA. The findings highlight the complex interplay between nitrosative stress, inflammation, and migraine severity, emphasizing the need for targeted therapeutic strategies that address these underlying mechanisms to improve patient outcomes and quality of life. Therapeutic strategies aimed at enhancing IPA levels or mimicking its effects could potentially mitigate the severity and frequency of migraine attacks. Future clinical trials are warranted to explore the efficacy of IPA supplementation or similar compounds in reducing migraine burden, which could lead to more effective and targeted treatments for patients suffering from this debilitating condition.

## Figures and Tables

**Table 1:** Comparison of demographic and biochemical results between patients and control group.

	Control	Patient	*p*
	(*n* = 30)	(*n* = 57)	
Age, years	29 (8)	33 (18.5)	0.555
Gender (F), *n*, (%)	24 (32.9)	49 (67.1)	0.544
BMI	24.15 (5.7)	26.23 (7.85)	0.565
IPA, ng/mL	7.4 ± 1.41	8.39 ± 1.66	**0.007**
NO^•^, umol/L	15 (2.68)	14.67 (3.68)	0.968
NOS, U/mL	35.81 ± 7.32	37.57 ± 8.77	0.353
ONOO^−^, umol/L	200.64 (17.27)	205.04 (28.93)	**0.038**

Abbreviations: F = Female, BMI = Body Mass Index, IPA = Indole-3-propionic acid, NO^•^ = Nitric Oxide, NOS = Nitric Oxide Synthase, ONOO^−^ = Peroxynitrite.

**Table 2:** Comparison of demographic and biochemical results between the groups in subgroup analysis.

	Control	EM	CM	*p*
	(*n* = 30)	(*n* = 28)	(*n* = 29)	
Age,	29 (8)	32 (19.25)	33 (18.5)	0.824
Gender (F), *n* (%)	24 (32.9)	22 (30.1)	27 (37)	0.253
BMI	24.15 (5.7)	26.25 (7.8)	25.48 (7.82)	0.645
IPA, ng/mL	7.4 ± 1.41	8.91 ± 1.63	7.88 ± 1.56	**0.001**
NO^•^, umol/L	15 (2.68)	13.7 (3.22)	16.61 (5.07)	**0.001**
NOS, U/mL	35.81 ± 7.32	35.91 ± 9.3	39.17 ± 8.07	0.225
ONOO^−^, umol/L	200.64 (17.27)	204.6 (34.43)	206.36 (24.31)	0.102

Abbreviations: EM = Episodic Migraine, CM = Chronic Migraine, F = Female, BMI = Body Mass Index, IPA = Indole-3-propionic acid, NO^•^ = Nitric Oxide, NOS = Nitric Oxide Synthase, ONOO^−^ = Peroxynitrite.

**Table 3:** Comparison of demographic and biochemical results between patients with EM and CM in subgroup analysis.

	EM	CM	*p*
	(*n* = 28)	(*n* = 29)	
Age, years	32 (19.25)	33 (18.5)	0.774
Gender (F), *n* (%)	22 (44.9)	27 (55.1)	0.144
BMI	26.43 ± 5.19	25.36 ± 4.35	0.401
Attack Frequency (monthly)	1 (0)	7 (3)	<0.001
Painful Days (monthly)	2 (2)	15 (1)	<0.001
Number of NSAIDs (monthly)	1 (2)	10 (5)	<0.001
VAS	6 (2)	7 (1)	0.008
HIT-6	60.5 (5.75)	65 (6.5)	0.019
MIDAS	1 (1)	4 (0)	<0.001
24 h QoL	76.5 (6)	41 (14)	<0.001
MMSE	29 (2.75)	28 (2.5)	0.063
Mig-SCog	4.64 ± 2.75	6.62 ± 2.29	0.005
IPA, ng/mL	8.91 ± 1.63	7.88 ± 1.56	0.018
NO^•^, umol/L	13.7 (3.22)	16.61 (5.07)	<0.001
NOS, U/mL	35.91 ± 9.3	39.17 ± 8.07	0.170
ONOO^−^, umol/L	204.6 (34.43)	206.36 (24.31)	0.523

Abbreviations: EM = Episodic Migraine, CM = Chronic Migraine, F = Female, BMI = Body Mass Index, NSAID = Non-Steroid Anti-Inflammatory Drug, VAS = Visual Analog Score, HIT-6 = Headache Impact Test 6, MIDAS = Migraine Disability Assessment Test, 24 h QoL = 24 h Quality of Life, MMSE = Mini-Mental State Examination, Mig-SCog = Migraine Attacks–Subjective Cognitive Impairments Scale, IPA = Indole-3-propionic acid, NO^•^ = Nitric Oxide, NOS = Nitric Oxide Synthase, ONOO^−^ = Peroxynitrite.

## Data Availability

The data analyzed in this research can be provided by the corresponding author upon a reasonable request.
